# Recurrence Leiomyosarcoma of the Popliteal Vein: A Rare Soft Tissue Sarcoma

**DOI:** 10.1155/2023/2788584

**Published:** 2023-02-16

**Authors:** Thanate Poosiripinyo, Sermsak Sukpanichyingyong, Krits Salang, Chat Sumananont, Thanapon Chobpenthai

**Affiliations:** ^1^Department of Orthopaedics, Khon Kaen Hospital, Khon Kaen, Thailand; ^2^Department of Orthopaedics, Srinagarind Hospital, Faculty of Medicine, Khon Kaen University, Khon Kaen, Thailand; ^3^Princess Srisavangavadhana College of Medicine, Chulabhorn Royal Academy, Bangkok, Thailand

## Abstract

**Background:**

Leiomyosarcoma (LMS) is a soft tissue sarcoma that originates from smooth muscle cells and constitutes approximately 5–10% of all soft tissue sarcomas. Vascular LMS is the least common subtype of LMS. About one-third of vascular LMS is located in the extremities, most commonly in the saphenous vein (25%). Vascular LMS originating from the popliteal vein is very rare, and to the best of our knowledge, only nine cases have been reported to date. *Case presentation*. We herein report a case of a 49-year-old woman who presented with recurrence of a mass that was located at the posterior aspect of the right proximal leg and extended to the popliteal fossa. She had mild pain and intermittent claudication without a history of an edematous leg. The tissue diagnosis was LMS. Wide en bloc resection of the tumor, including the segment of the involved popliteal vein, was performed without venous reconstruction. The patient received no other adjuvant treatments. At the 16-month follow-up, she had good oncologic and functional outcomes.

**Conclusion:**

Vascular LMS at the popliteal vein is uncommon but should be considered as a differential diagnosis in a patient who presents with a mass at the popliteal fossa. The magnetic resonance imaging (MRI) and core needle biopsy were needed for a definite diagnosis. The mainstay of treatment is wide en bloc resection of the tumor, including the involved segment of the vein. Venous reconstruction after resection is unnecessary in chronic cases without a history of an edematous leg. Radiotherapy is an important adjuvant for local control when the surgical margins are close or positive. The role of chemotherapy in systemic management remains unclear.

## 1. Introduction

Sarcoma is a malignant tumor that arises from mesenchymal cells and constitutes approximately 1% of all malignancies in adults. About 510% of all soft tissue sarcomas are leiomyosarcoma (LMS). LMS is a soft tissue sarcoma that originates from smooth muscle cells, and it can be divided into three subtypes according to the site of presentation: soft tissue LMS, cutaneous LMS, and vascular LMS [1].

Vascular LMS is the least common subtype of LMS. The first case of vascular LMS was reported by Perl in 1871, and the tumor originated from the inferior vena cava [2–4]. About one-third of vascular LMS is located in the extremities, most commonly in the saphenous vein (25%) [1]. Vascular LMS originating from the popliteal vein is very rare, and to the best of our knowledge, only nine cases have been reported to date [2–7].

A definitive diagnosis of vascular LMS is made by tissue biopsy and immunohistochemistry studies. The mainstay of treatment is en bloc resection of the tumor, including the involved segment of the vein, with a wide surgical margin [1, 4, 7]. Venous reconstruction after resection is not necessary in every case (especially in chronic cases with no history of an edematous leg) depending on the collateral venous circulation drainage [6]. Radiotherapy is an important adjuvant treatment for local control when the surgical margins are close or positive [1, 7]. The role of chemotherapy for systemic control remains unclear.

We herein report a case of popliteal LMS and present a literature review of previously reported cases. Although the frequency of this type of tumor is low, it should be considered in a patient who presents with a mass at the popliteal fossa and intermittent claudication.

## 2. Case Presentation

**Figure 1:**
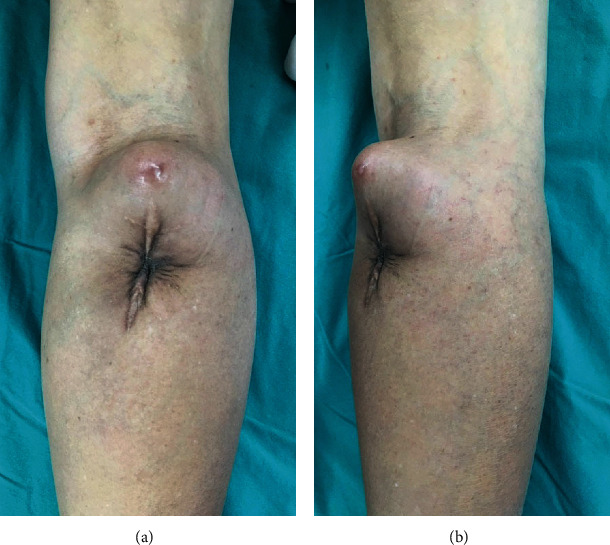
Physical examination findings. (a) Posterior view. (b) Oblique view.

A 49-year-old woman presented with a soft tissue mass at the posterior aspect of the right proximal leg in 2019. Tumor removal was performed by the general physician without tissue pathologic examination or evaluation of the surgical margin. One year later, she returned with a recurrent mass at the same site of the previous surgery. The mass had exhibited slow growth. She had mild pain and intermittent claudication without a history of an edematous leg. Physical examination revealed an ill-defined mass that was located at the posterior aspect of the right proximal leg and extended to the popliteal fossa. It was 10 × 10 cm in size, movable, and mildly tender (Figures 1(a) and 1(b)).

**Figure 2:**
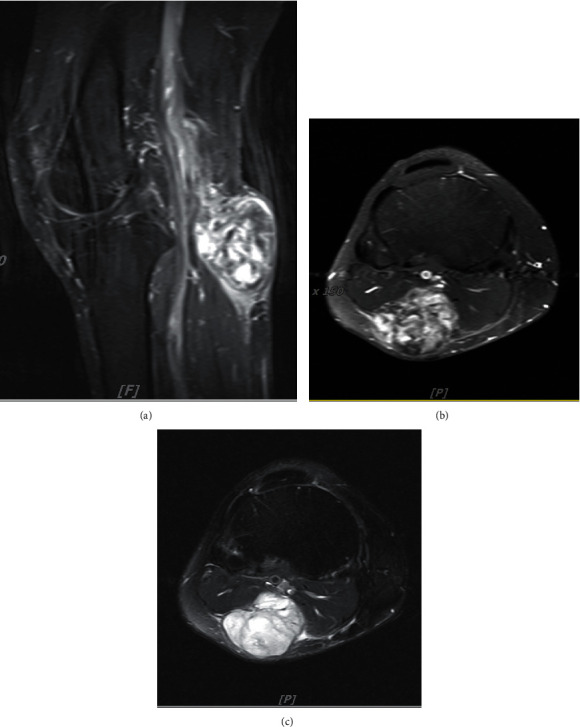
Magnetic resonance imaging. (a) Sagittal view with gadolinium contrast. (b) Axial view with gadolinium contrast. (c) Axial T2-weighted image.

Plain radiographs revealed a soft tissue mass on the posterior aspect of the right proximal leg immediately distal to the popliteal fossa without any bone lesions. Magnetic resonance imaging (MRI) showed a deep-seated heterogeneous soft tissue mass originating from the popliteal vein (Figures 2(a), 2(b), and 2(c)).

**Figure 3:**
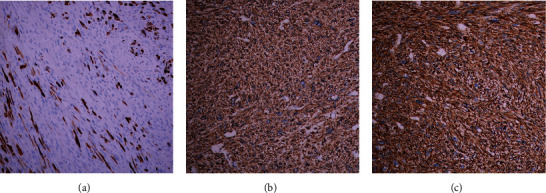
There is positive immunoreactivity for (a) desmin, (b) smooth muscle actin, and (c) caldesmon, which supports the diagnosis of leiomyosarcoma.

Chest computed tomography (CT) for staging revealed no pulmonary metastasis. A core needle biopsy was performed. The pathological report indicated a malignant soft tissue tumor that was immunohistochemically positive for smooth muscle actin, epithelial membrane antigen, vimentin, caldesmon, and desmin, compatible with LMS (Figures 3(a), 3(b), and 3(c)).

**Figure 4:**
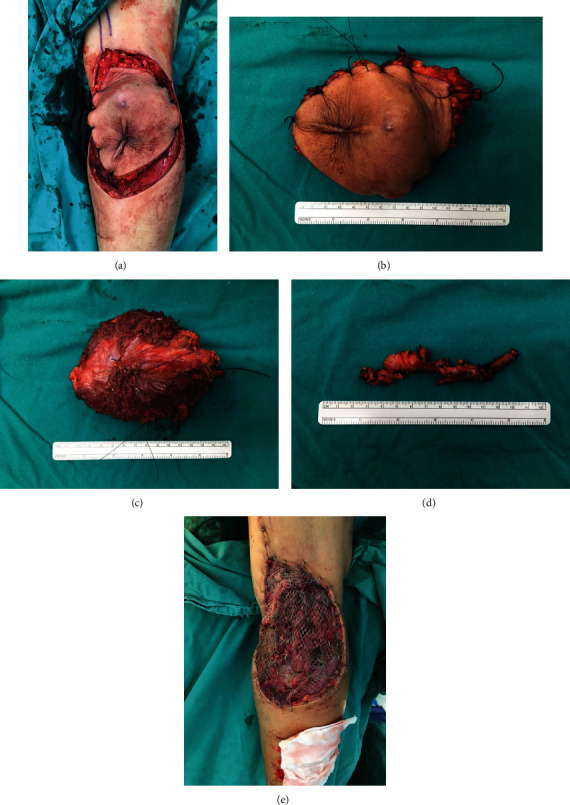
Tumor removal. (a) Incision. (b) Tumor after en bloc excision (superficial view). (c) Tumor after en bloc excision (deep margin). (d) Popliteal vein involved by tumor. (e) Surgical wound covered by split-thickness skin graft.

The patient underwent wide en bloc resection of the tumor, including the segment of the involved popliteal vein, without venous reconstruction (Figures 4(a), 4(b), 4(c), 4(d), and 4(e)).

**Figure 5:**
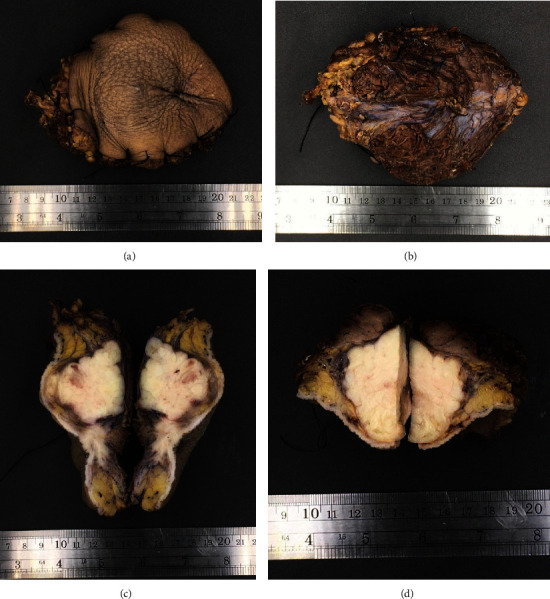
Gross specimens. (a) Posterior part. (b) Anterior part. (c) Medial section (saggital plane). (d) Superior section (transverse plane).

The surgical wound was covered by a split-thickness skin graft. The final pathology report from the surgical specimen revealed a typical intersecting 90-degree angle fascicular arrangement of spindle cells as well as scattered pleomorphic hyperchromatic cells with typical cigar-shaped nuclei and eosinophilic cytoplasm, compatible with LMS (Figures 5(a), 5(b), 5(c), and 5(d)).

**Figure 6:**
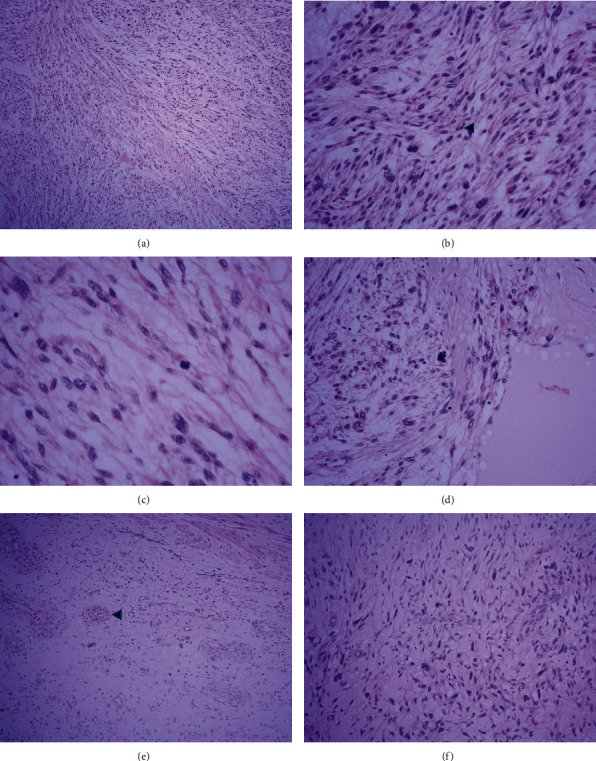
(a) Low power magnification of leiomyosarcoma reveals typically intersecting 90-degree angle fascicular arrangement of spindle cells. (b) High magnification area shows scattered pleomorphic hyperchromatic cells with typically “cigar-shaped” nuclei (arrowhead) and eosinophilic cytoplasm. (c) and (d) Reveals increase mitotic activity with atypical forms. (e) and (f) Sections from the main vessel show the neoplasm derived from the tunica media of the vessel (arrowhead) and high magnification reveals significantly cytologic atypia.

All resected margins were free of tumor tissue (Figures 6(a), 6(b), 6(c), 6(d), 6(e), and 6(f)).

**Figure 7:**
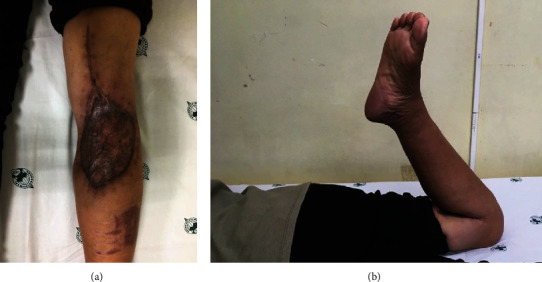
Clinical result at the 16-month follow-up. (a) Good healing of the surgical wound was attained without tumor recurrence. (b) Full range of motion of the involved knee was attained.

The patient received no other adjuvant treatments. The postoperative course was uneventful, and the patient was discharged within 1 week of surgery. No complications, adverse outcomes, local recurrence, or distant metastasis was reported at 16 months after surgery. The patient had good functional outcomes (Figures 7(a) and 7(b)).

## 3. Discussion

Vascular LMS is the least common subtype of LMS and originates from the tunica media of the vessel wall [1]. The popliteal vein is a very uncommon location of vascular LMS, and to the best of our knowledge, only nine cases have been reported to date [2–7].

The clinical presentation of vascular LMS of the popliteal vein includes a mass at the popliteal fossa, intermittent claudication, and pain with varying degrees of intensity. Some patients' presentation may mimic deep vein thrombosis (DVT), but patients with DVT usually have risk factors for DVT [3]. In an otherwise healthy patient who presents with intermittent claudication and leg edema without risk factors for DVT, a vascular tumor or tumor that compresses and obstructs the venous circulation should be considered, although such tumors are very rare [3, 4, 8]. The mass can grow rapidly and involve adjacent structures such as bone, nerves, and other vessels. Affected patients usually have a history of leg edema because of the obstruction of venous return, but some patients have no history of an edematous leg because of the development of collateral circulation, especially patients with a slowly growing mass. Basu et al. [6] reported a case of a 35-year-old woman with LMS of the left popliteal vein without leg edema. The tumor was resected without vascular reconstruction. Killoran et al. [3] and Karamoshos et al. [4] reported cases of LMS of the popliteal vein in patients with a history of leg edema. En bloc resection of the popliteal vein tumor with vascular reconstruction was performed in both cases. In our case, the patient presented with recurrence of a slowly growing mass at the right popliteal fossa and no history of leg edema. She underwent wide en bloc resection of the tumor, including the segment of the involved popliteal vein, without venous reconstruction. She had good oncologic and functional outcomes without complications.

MRI is the imaging technique of choice to identify tumor characteristics and determine the tumor size, extension, and anatomic relationships [3, 9]. MRI is also very useful for preoperative planning. However, MRI is not useful in differentiating benign from malignant lesions. CT angiography (CTA) is also helpful to identify intraluminal tumors and their collateral circulation for preoperative planning [3, 5]. In patients with adequate collateral circulation, wide resection without vascular reconstruction is suitable. In our case, we did not use CTA for preoperative planning because it was not available at that time. We found that the popliteal vein was totally occluded by the tumor intraoperatively, but the patient had no history of leg edema. Therefore, we considered that she already had an adequate collateral circulation. We considered performing wide en bloc resection without vascular reconstruction. Basu et al. [6] also reported a case of a 35-year-old woman with LMS of the left popliteal vein without leg edema, and the investigation did not include CTA. The patient underwent wide resection without vascular reconstruction.

The definitive diagnosis of LMS is achieved by histopathology and immunohistochemistry. The histopathological study of vascular LMS is similar to that of LMS at any location. The findings are characterized by pleomorphic hyperchromatic spindle-shaped cells with typical cigar-shaped nuclei and eosinophilic cytoplasm [1, 10]. Immunohistochemical studies are very helpful for a definitive diagnosis; they are usually positive for smooth muscle actin, vimentin, desmin, calponin, and smooth muscle myosin heavy chains and negative for S-100, inhibin alpha, and CD117 [1].

The mainstay of treatment is wide en bloc resection of the tumor, including the involved segment [1, 4, 7]. Venous reconstruction after resection is not necessary in patients who have no history of leg edema and in whom CTA reveals collateral circulation [6]. Radiotherapy is an important treatment for local control when surgical margins are positive or close [1, 7]. The efficacy of chemotherapy for systemic control is still unclear. In our case, we performed wide en bloc resection of the tumor, including the segment of the involved popliteal vein, without venous reconstruction. The patient did not receive radiotherapy or chemotherapy because the surgical margin was negative and there was no evidence of metastasis.

The 5-year survival rate of vascular LMS is about 32% [3, 8], and approximately 10% of patients present with lung or liver metastasis at the time of diagnosis because the vascular tumors have direct access to the blood circulation [3, 11]. Vascular LMS generally has a poor prognosis.

We have summarized the clinical data of nine patients with LMS of the popliteal vein from our literature review in Table 1.

## 4. Conclusion

Vascular LMS at the popliteal vein is uncommon but should be considered as a differential diagnosis in a patient who presents with a mass at the popliteal fossa. The mainstay of treatment is wide en bloc resection of the tumor, including the involved segment of the vein. Venous reconstruction after resection is not necessary in chronic cases without a history of an edematous leg.

## Figures and Tables

**Table 1:** Summarized clinical data of nine patients with leiomyosarcoma of the popliteal vein.

Case no.	Year of report	Sex	Age (years)	Size (cm)	History of leg edema	Lung metastasis	Treatment	Margin	Adjuvant treatment	F/U
1	1979	M	66	6 × 5 × 5	NA	Yes	AK amputation	Negative	Postop XRT	3-Year F/U, alive with lung metastasis
2	1984	M	63	18 × 8 × 7	NA	Yes	AK amputation	Negative	ND	3-Month F/U, death
3	1987	M	67	NA	Yes	No	AK amputation	Negative	Postop CMT	2-Year F/U, no recurrence
4	1988	F	35	9.5 × 6 × 6	No	Yes	Excision without vascular reconstruction	Positive	Postop XRT and CMT	3-Month F/U, alive with lung metastasis
5	2003	M	62	8.5 × 5.7 × 3	Yes	NA	Excision with vascular reconstruction	Negative	Postop XRT	10-Month F/U, no recurrence
6	2005	F	68	7 × 4 × 3	Yes	No	Excision with vascular reconstruction	Negative	ND	18-Month F/U, no recurrence, no metastasis
7	2009	F	54	3	No	No	NA	NA	NA	NA
8	2009	M	77	NA	Yes	No	NA	NA	NA	NA
9	2009	F	63	13	No	No	NA	NA	NA	NA

## Data Availability

The datasets used or analyzed during the current study are available from the corresponding author upon reasonable request.
